# Optimizing Trap Characteristics to Monitor the Leaffooted Bug *Leptoglossus* *zonatus* (Heteroptera: Coreidae) in Orchards

**DOI:** 10.3390/insects11060358

**Published:** 2020-06-09

**Authors:** Houston Wilson, Jessica J. Maccaro, Kent M. Daane

**Affiliations:** 1 Department of Entomology, University of California, Riverside, Riverside, CA 92521, USA; jessica.maccaro@ucr.edu; 2 Department of Environmental Science, Policy and Management, University of California, Berkeley, Berkeley, CA 94720-3114, USA; kdaane@ucanr.edu

**Keywords:** leaffooted bug, cross-vane panel trap, trap color, *Leptoglossus*

## Abstract

The leaffooted bug, *Leptoglossus* *zonatus* (Heteroptera: Coreidae), has become a key pest of almonds, pistachios, and pomegranates in California. Adults and nymphs directly feed on nuts and fruits, which reduces crop yield and quality and can facilitate pathogen infections. Current monitoring strategies require growers to actively sample the tree canopy, with no economic thresholds being developed for this pest. To improve monitoring of *L.* *zonatus*, a three-year study was conducted to identify an optimal trap. A hanging cross-vane panel trap was identified as the best trap type in Year 1, and subsequent work in Years 1–3 focused on refining its use by modifying surface texture and color. Results indicated that coating trap surfaces with the lubricant fluon improved trap catching ability, and adults were most frequently recovered in yellow traps. A hanging cross-vane panel trap with these features could serve as the basis for the development of a new monitoring system for this pest in orchards, which could be improved further if semiochemical lures will be developed.

## 1. Introduction

The genus *Leptoglossus* is neotropical in origin, with most species limited to Central and South America [1,2], where they are considered pests for a wide variety of crops [3,4,5,6,7,8]. In North America, the key economic species are *L. clypealis*, *L. occidentalis, L. phyllopus,* and *L. zonatus*. *Leptoglossus occidentalis* primarily attack coniferous trees [9,10], whereas the other three species have been recovered from a range of perennial crops that include tree nuts, citrus, peaches, and pomegranates [11,12,13,14]. The primary agricultural pest species in California are *L. clypealis* and *L. zonatus*, which are known to attack almonds, pistachios, and pomegranates when not feeding on a variety of weedy annual species [15]. Early reports first documented *L. clypealis* feeding on pistachio, which led to nut drop and epicarp lesion [16,17]. While historically *L. clypealis* has been the dominant species found on California tree nuts [18], recent surveys have noted a shift towards *L. zonatus*, which is now considered the primary species attacking these crops [15,19,20].

These species of *Leptoglossus* overwinter as adults in aggregations in sheltered areas, such as evergreen trees, shrubs, and residential structures, and in the spring disperse in search of food and reproduction sites [10,14,15,21]. In California, *L. clypealis* and *L. zonatus* complete three generations, but a fourth generation is possible if quality food sources and mild fall-winter temperatures are present [19]. While there are many plant species these leaffooted bugs will feed on, adults typically begin to attack almonds in April–May. As almond shells harden, adults (either from the overwintering or first summer generation) move over to pistachios in May–June, which are still vulnerable during this period. Similarly, as pistachio shells harden, the later summer generation(s) of adult leaffooted bug shift to pomegranates in August–September, and typically complete a generation while residing in this crop until late November, at which point adults move to overwintering aggregation sites. While the most extensive crop damage is due to adults, who have strong mouthparts and can disperse widely, feeding by developing nymphs can also have an impact on crop yield and quality.

Current recommendations for monitoring leaffooted bugs include beat sampling the tree canopy, visually searching trees for adults, or assessing immature nuts for signs of feeding damage [15,21]. All of these approaches are very time and labor intensive, and no economic thresholds associated with any of these sampling methods exist. Furthermore, damaging populations of *L. zonatus* adults can be sporadic and tend to arrive rapidly, which makes them difficult to predict and/or detect in a timely manner. As such, use of chemical controls is typically based on presence/absence of *Leptoglossus* spp. and largely rely on pyrethroids, like bifenthrin and lambda-cyhalothrin. Design of an effective trap and lure system for monitoring *L. zonatus* could improve sampling efficiency and facilitate the development of economic thresholds. Here, as a first step towards developing this type of system, different trap types were screened for their ability to catch *L. zonatus* under field conditions, with parallel optimization of several trap parameters.

## 2. Materials and Methods

A series of field studies were carried out over a three-year period in the San Joaquin Valley, California. In an initial study in Year 1, the efficacy of different trap types was compared, in order to identify the most promising one. The subsequent efforts in Years 1–3 focused on refining the use of the most efficient trap, by modifying trap color and surface coating. Working with unbaited traps required that all studies took place at sites with heavy infestations in the late summer and fall when *L. zonatus* populations are highest.

### 2.1. Trap Comparison Study

Five trap types and three bait treatments were evaluated at three field sites that included an olive, pistachio, and pomegranate orchard, each heavily infested with *L. zonatus*. The olive orchard was 2.8 ha (7 ac), the pistachio orchard was 0.8 ha (2 ac), and the pomegranate orchard was arranged as a hedge that was 4.6 m (15 ft) wide by 419.4 m (1376 ft) long.

Trap types included a black hanging cross-vane panel trap (Intercept Trap, Alpha Scents, West Linn, OR, USA), a 1.2 m (4 ft) tall black pyramid trap (Dead Inn, AgBio Inc., Denver, CO, USA), a 0.6 m (2 ft) tall black pyramid trap (Dead Inn, AgBio Inc., Denver, CO, USA), a 15.2 × 30.5 cm (6 × 12 in) clear sticky dual panel trap (Pherocon, Trécé Inc., Adair, OK, USA), and a green-white-yellow bucket trap (Multi-color UNI-Trap, Alpha Scents, West Linn, OR, USA). The collection buckets of the hanging cross-vane panel traps and bucket traps contained 443 mL (15 oz) of a killing solution, which consisted of 9.9 mL (2 tsp) biodegradable detergent diluted in 3.8 L (1 gal) of water.

Trap baits included split pomegranate (50 g), almond meal (40 g) mixed with crude almond oil (10 g), and a no bait control. Baits were placed in 10.2 × 15.2 cm (4 × 6 in) organdy mesh bags. These bait bags were then suspended from the center of the hanging panel trap, placed inside the collection cup at the top of the pyramid traps, attached with a binder clip to the clear sticky trap, or suspended in the collection cup inside the bucket trap. In total there were 15 unique trap × bait treatment combinations.

At each of the three sites, a randomized complete block design was used with five replicates per site. Replicates were spaced 36.6 m, 18.3 m, and 10 m apart and within replicates the different trap/bait treatments were spaced 9.1 m, 5.2 m, and 33 m apart at the olive, pistachio, and pomegranate sites, respectively. Traps were set up and monitored weekly between 7 September–18 October in Year 1. Each week the baits and collecting solution were replaced and all adult *Leptoglossus* spp. were removed, sexed, and identified to species.

### 2.2. Surface Coating Study

In a follow-up study, the use of fluon (Insect-a-Slip, BioQuip, Rancho Dominguez, CA, USA) was evaluated to improve recovery of *Leptoglossus* spp. in the black hanging cross-vane panel traps. There were five treatments that included a coating of undiluted fluon; dilutions of 50%, 25%, and 12.5% fluon; and a control trap with no fluon. In each fluon treatment, a single layer of solution was painted over all trap surfaces. No baits were used with any of the traps. Traps were evaluated using a randomized complete block design with five replicates at the pomegranate site described above. Traps were set up and monitored weekly from 13 November–4 December in Year 1. As before, each week, the collecting solution was replaced and all adult *Leptoglossus* spp. were removed, sexed, and identified to species.

### 2.3. Trap Color

The effect of the hanging cross-vane panel trap color was evaluated each fall in a heavily infested pomegranate orchard over a three-year period. Black traps were compared to red, yellow, green, blue, and white traps. Field studies utilized a randomized complete block design with five replicates. In Years 1–2, this study was located at the pomegranate site described above and took place during 4–18 December, in Year 1, and 22 August–20 November, in Year 2. In Year 3, the study took place 27 September–3 December in a 0.4 ha (1 ac) pomegranate orchard at the UC Kearney Agricultural Research and Extension Center, Parlier, California, USA. Replicates were spaced 10 m and 10.3 m apart and traps within replicates were spaced 3 m and 9.1 m apart, in Years 1–2 and 3, respectively.

### 2.4. Statistical Analysis

Data on insect abundance from each trial were log (x + 1) transformed and analyzed with generalized linear mixed-models using the “glmer” function in the “lme4” package in the R statistical program (http://www.r-project.org/). Fixed effects were evaluated through model comparison using chi-square tests via the “drop1” function. When a multilevel categorical variable was found to be significant, means were separated using post hoc Tukey contrasts (“glht” function in the “multcomp” package). Male and female *L. zonatus* were evaluated separately.

For the trap/bait study, fixed effects included “Trap Type” and “Bait,” with the random effects “Replicate Block” nested within “Site” within “Sample Week.” Replicate Block was included as a random effect, since each block contained multiple repeats of the different trap types and baits. An interaction term for “Trap Type × Bait” was initially evaluated, but since it was found to be non-significant, the analysis presented here models these factors separately. The fluon study included fixed effect “Fluon Dilution” and the random effect “Sample Week.” Finally, the trap color study included fixed effect “Trap Color,” with random effects “Site” nested within “Sample Week” within “Year.”

## 3. Results

**Figure 1:**
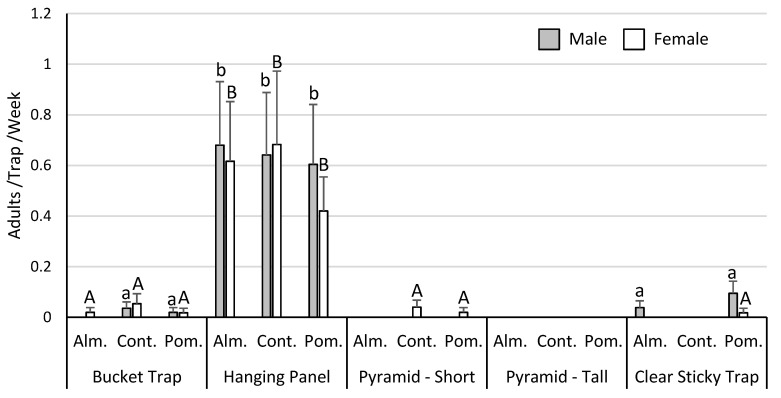
The hanging cross-vane panel trap consistently captured the most *L. zonatus*, regardless of bait type. Columns of the same color without a common letter differ significantly. Alm. = almond meal bait, Cont. = no bait control, and Pom. = pomegranate bait; Bucket Trap = green-white-yellow bucket trap, Hanging Panel = black hanging cross-vane panel trap, Pyramid - Short = 0.6 m pyramid trap, Pyramid - Tall = 1.2 m pyramid trap, and Clear Sticky Trap = clear sticky dual panel trap.

**Figure 2:**
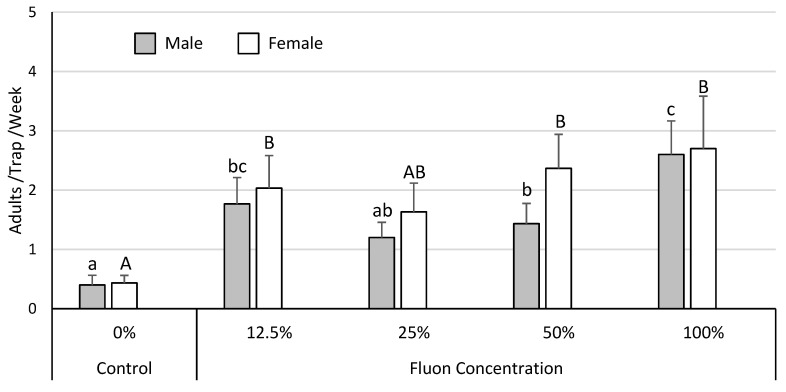
The addition of fluon increased trap capture of *L. zonatus.* Columns of the same color without a common letter differ significantly.

**Figure 3:**
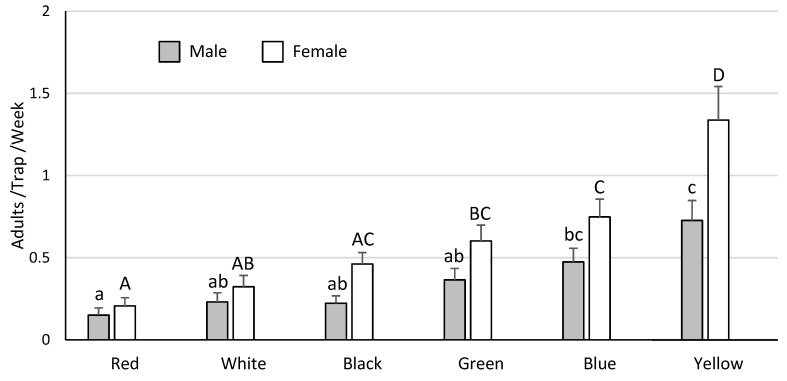
Yellow traps captured the most *L. zonatus*, followed by blue and green traps. X-axis defines the color of trap evaluated. Columns of the same color without a common letter differ significantly.

Almost all the *Leptoglossus* spp. recovered in these trials were *L. zonatus*, with *L. clypealis* rarely encountered. As such, all of the data analyses focused on *L. zonatus* alone. Analysis of the trap and bait trial data (*n* = 400, total *L. zonatus* females = 103, males = 114) indicated that capture of *L. zonatus* was influenced by trap type (males χ^2^ = 106.8, *p* < 0.001; females χ^2^ = 101.0, *p* < 0.001), but not by bait (males χ^2^ = 0.2, *p* = 0.91; females χ^2^ = 0.2, *p* = 0.92). The hanging cross-vane panel trap captured the most *L. zonatus* adults (Figure 1). In the surface treatment trial (*n* = 75, total *L. zonatus* females = 277, males = 213), *L. zonatus* catch was increased substantially by coating trap surfaces with fluon (males χ^2^ = 33.6, *p* < 0.001; females χ^2^ = 20.4, *p* < 0.001), even when highly diluted (Figure 2). Finally, data from the multi-year trap color experiment (*n* = 568, total *L. zonatus* females = 342, males = 201) indicated differences in *L. zonatus* catch across the different trap colors (males χ^2^ = 40.6, *p* < 0.001; females χ^2^ = 70.0, *p* < 0.001). Yellow traps were the most attractive, followed by blue and green traps, while white and red traps were the least attractive (Figure 3).

## 4. Discussion

This series of experiments demonstrated that an unbaited hanging cross-vane panel trap is attractive to *L. zonatus*, especially a yellow trap. Capture can be increased by coating trap surfaces with fluon to reduce insect ability to grip the trap surface. Inclusion of a split pomegranate or almond meal bait did not increase trap catch, indicating that these materials have low attractive ability on this insect species, and that *L. zonatus* apparently are attracted to the hanging cross-vane panel trap itself. Response of *L. zonatus* to a large dark object, such as this panel trap, is similar to observations by Panizzi [22], who reported that *L. zonatus* aggregated on unbaited plastic cylinder traps when the cylinders were first introduced into corn fields.

The cross-vane panel trap was initially developed to monitor Coleoptera in forests [23,24], as a replacement or supplement for the multiple-funnel trap [25]. Subsequent work with the cross-vane panel trap demonstrated its utility for trapping cerambycid and buprestid beetles [26,27,28]. Trapping efficacy for beetle species can be further enhanced, sometimes more than ten-fold, by coating trap surfaces with fluon or other lubricants [29,30,31]. Our results demonstrated analogous increases in trapping efficiency when traps were treated with fluon.

Cross-vane panel traps have also been used to effectively trap Hemipterans, such as triatomines (Reduviidae: Triatominae) [32], bagrada bug (Pentatomidae: *Bagrada hilaris*) [33], and brown marmorated stink bug (Pentatomidae: *Halyomorpha halys*) [34], but apparently are not well suited for spotted lanternfly (Fulgoridae: *Lycorma delicatula*) [35]. No traps specifically for *Leptoglossus* spp. have been developed. The scant literature includes a prototype bottle trap for *L. zonatus* [36], along with a study that mentions the use of multi-funnel traps for testing *L. occidentalis* attraction to caged aggregations of males and females [37]. Bycatch of non-target organisms in the cross-vane panel trap was minimal, with some honeybees (Apidae: *Apis mellifera*) recovered in blue and green traps and assassin bugs (Reduviidae: *Zelus* spp.) in blue and yellow traps (data not shown).

The effect of trap color has been widely evaluated across multiple insect orders, including Coleoptera [38,39,40], Lepidoptera [41,42,43], Hymenoptera [44,45,46,47], and Hemiptera [48,49,50,51,52]. The only previous study to evaluate *Leptoglossus* spp. responses to color was a laboratory study, which found that *L. zonatus* adults and nymphs were primarily attracted to blue and green [53]. These results were partially complimented in our study, in which blue and green were the second and third most attractive colors after yellow. Response of *L. zonatus* to trap color in the current study may have been skewed by differences in trap temperature, which likely varied between the lighter and darker colored traps under field conditions. This may be an important consideration, given that a recent study of *L. occidentalis*, a relative species of *L. zonatus*, indicated that this species utilizes infrared cues to locate host plants [54]. It may be that *L. zonatus* utilizes infrared cues in a similar way. As such, subsequent field evaluations of trap color effects on *L. zonatus* could be improved by controlling for, or at least recording data on, trap temperature.

## 5. Conclusions

Identification of the hanging cross-vane panel trap for *L. zonatus* represents an important step forward in the development of an effective monitoring program for this pest, especially the highly mobile adult, and will allow for the future screening of candidate lures for this insect under field conditions [55]. Once paired with an attractive lure, studies will need to determine the most effective density and spatial arrangement of traps to accurately reflect orchard populations of *L. zonatus* and correlate trap captures with economic thresholds. If perfected, this type of trap and lure sampling system will reduce the sampling effort and lead to earlier and/or more accurate detection of *L. zonatus* populations in orchards. It is also possible that these traps could work well for other *Leptoglossus* pest species, such as *L. occidentalis*, which has recently invaded Europe, and may be worth further investigation in that context [56,57].
